# Identification of metabolic vulnerabilities and synthetic lethal targets utilizing artificial intelligence-driven precision medicine

**DOI:** 10.3389/fonc.2026.1972698

**Published:** 2026-10-27

**Authors:** Rashid Mir, Manal Aljammaz, Amr Ammar Alsufyani, Yahya Ali Khubrani, Aziz Dhaher Albalawi, Shouq S. Alsubaie, Wed Albalawi, Naseh A. Algehainy, Mohammed M. Jalal, Malik A. Altayar, Reema M. Almotairi, Faris J. Tayeb, Ruqaiah I. Bedaiwi, Faisal H. Altemani, Kholoud S. Almasoudi, Ibrahim Altedlawi Albalawi, Aisha Ali Awaji

**Affiliations:** 1 Prince Fahad Bin Sultan Chair for Biomedical Research, University of Tabuk, Tabuk, Saudi Arabia; 2 Molecular Medicine, Department of Medical Laboratory Technology, Faculty of Applied Medical Sciences, University of Tabuk, Tabuk, Saudi Arabia; 3 Department of Medical Laboratory Technology,Faculty of Applied Medical Sciences, University of Tabuk, Tabuk, Saudi Arabia; 4 Department of Surgical Oncology, Faculty of Medicine, University of Tabuk, Tabuk, Saudi Arabia; 5 Department of Biology,Faculty of Science, University of Tabuk, Tabuk, Saudi Arabia

**Keywords:** artificial intelligence, cancer metabolism, metabolic vulnerabilities, multi-omics integration, precision oncology, single-cell metabolomics, synthetic lethality, systems biology

## Abstract

Cancer metabolism is highly plastic, and most attempts to turn that plasticity into a therapeutic target fail for a specific, recurring reason: a computational prediction of a metabolic vulnerability is not the same evidentiary object as an experimentally confirmed one, and the two are routinely discussed as if they were interchangeable. This review takes that distinction as its starting point rather than its conclusion. Artificial intelligence and genome-scale systems biology have made it easy to generate metabolic hypotheses at a scale no experimental program could match — machine learning models trained on genomic, transcriptomic, proteomic, and metabolomic data now output candidate vulnerabilities, biomarkers, and drug combinations faster than they can be tested — but the resulting literature has not been consistently honest about how far any given prediction has actually traveled toward clinical relevance. We examine six areas where this gap is most visible: multi-omics data integration, genome-scale metabolic network modeling, computational discovery of metabolic vulnerabilities and synthetic lethal interactions, single-cell and spatial resolution of metabolic heterogeneity, AI-guided drug and biomarker discovery, and the small set of studies that have actually reached clinical testing. Rather than summarizing these areas as separate literatures, we grade every substantive finding discussed against an explicit four-tier evidence standard — associative, mechanistic, perturbation-validated, or clinically tested — and we apply that standard critically rather than deferentially: several widely cited computational vulnerabilities remain unconfirmed outside the cell lines in which they were discovered, genome-scale models continue to carry unresolved gaps in reaction coverage that limit their predictive reach, and, of the AI-derived metabolic predictions discussed in this review, only one has so far been tested in a completed prospective clinical trial. We argue that this shortage of prospective validation, not a shortage of computational sophistication, is the field’s central constraint, and we close by identifying where methodological effort would do the most good: standardized validation reporting, tissue-resolved rather than bulk metabolic models, and closer coupling between computational prediction and the specific experimental designs capable of falsifying it.

## 1 Introduction

Otto Warburg’s observation that cancer cells prefer lactate production even when oxygen is available, now known as the Warburg effect, was one of the earliest signs that altered metabolism is part of the disease, not just a by-product of it. The biology that has since emerged is much less tidy. Glycolysis often runs alongside glutamine catabolism through enzymes such as glutamic pyruvate transaminase 2, helping to supply the carbon and nitrogen needed for rapid proliferation (1). Oncogenic drivers such as KRAS and MYC can reshape glycolytic and glutaminolytic flux, and metabolic phenotypes differ not only across cancer types but also between tumors, and even between cells inside the same tumor (2). Kim and DeBerardinis (3) reviewed that variability in detail, and the practical lesson is simple: one metabolic signature is rarely enough.

**Figure 1** previews the evidence-tier framework used throughout this review to keep that gap visible rather than implicit.

**Figure 1:**
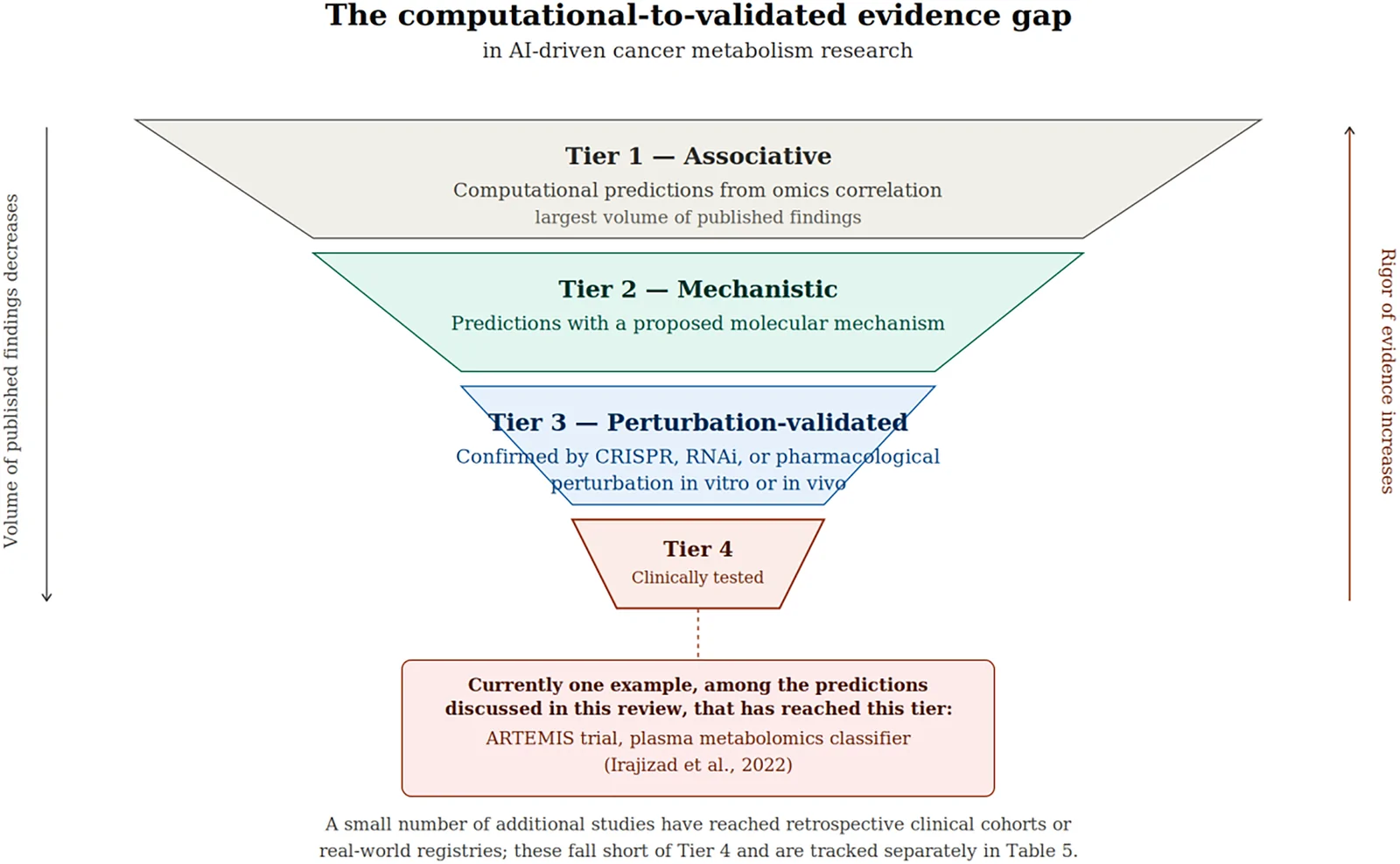
The computational-to-validated evidence gap in AI-driven cancer metabolism research.

A four-tier evidentiary pyramid, applied consistently across this review: associative findings from omics correlation (the largest volume), mechanistic findings with a proposed molecular basis, perturbation-validated findings confirmed by CRISPR, RNAi, or pharmacological manipulation *in vitro* or *in vivo*, and, narrowest, findings evaluated in a completed prospective clinical trial. Among the AI-derived metabolic predictions discussed in this review, only one — a plasma metabolomics classifier assessed within the ARTEMIS neoadjuvant trial in triple-negative breast cancer (4) — currently occupies the top tier; a small number of additional studies have reached retrospective clinical cohorts or real-world registries, which fall short of this standard and are tracked separately in **Table 1**.

**Table 1:** AI-based biomarker and prediction validation framework applied to metabolic cancer studies reviewed in this article.

Class	Validation requirement	Approximate status of reviewed studies	Illustrative example from this review
Class A	Concordance studies only (computational/analytical)	Majority of vulnerability prediction and multi-omics integration studies	(5) (regulatory-metabolic synthetic lethality, computational validation only)
Class B	Analytical validation (cell line or experimental confirmation)	Studies with experimental confirmation in cell lines or animal models	(6) (gMCS RRM1 prediction, 4 multiple myeloma cell lines validated)
Class C1	High-quality retrospective clinical or real-world trial data	Small number of studies with patient-level clinical data	(7) (morphometric biomarkers, NCT02430480, n=37 prostate cancer patients)
Class C2	Prospective clinical trial validation for treatment response prediction	Essentially absent in current metabolic AI literature; closest: ARTEMIS trial	(4) (AI metabolomics, ARTEMIS trial NCT02276443, TNBC)

A, concordance study; Class B, analytical validation; Class C1, retrospective clinical validation; Class C2, prospective clinical trial validation for treatment response prediction. Framework adapted from Ligero et al. (8). TNBC, triple-negative breast cancer; NCT, National Clinical Trialidentifier.

Genome-scale metabolic models were an early systems-level response to that complexity. By capturing the stoichiometry of thousands of reactions simultaneously, they recast the Warburg effect as a predictable consequence of cells optimizing biomass production under enzyme-capacity constraints rather than as an isolated curiosity (9). That reframing was genuinely useful, but it is worth being direct about what these models still cannot do. Even extensively curated genome-scale reconstructions carry substantial gaps in reaction coverage and rely on generic biomass objective functions that do not capture tissue- or tumor-specific stoichiometry, so that a flux prediction is only as reliable as the reconstruction’s completeness for the pathway in question, a limitation that model-comparison studies have documented repeatedly and that is too rarely foregrounded when these models are cited as evidence for a specific therapeutic target (10). The search for metabolic vulnerabilities has, as a result, shifted from individual genes toward broader network structure, but network-level predictions inherit network-level uncertainty, and that uncertainty does not disappear simply because the model is large.

Artificial intelligence has become the field’s other major pillar, and it changes the character of this uncertainty rather than resolving it. Genome-scale models supply mechanistic structure; machine learning and deep learning extract signal from the high-dimensional multi-omics data that sequencing and mass spectrometry platforms now generate at a pace no manually curated model can track (11). The result is a marked asymmetry: computational hypothesis generation has scaled by orders of magnitude, while the experimental and clinical infrastructure needed to test each hypothesis has not scaled at anything close to the same rate. This review’s organizing claim is that this asymmetry, rather than any single technical limitation of AI or systems biology individually, is the central problem the field now faces, and that the appropriate response is not simply to generate better predictions but to be far more disciplined about stating, for each one, how much evidence actually supports it. We track that discipline through a discovery loop, from exploratory signal-finding to mechanistic interpretation, perturbation-based validation, and clinical use, organized around six themes: data integration, network-level modeling, vulnerability identification, spatial and single-cell resolution, drug and biomarker discovery, and clinical translation.

Two recent contributions map adjacent territory, and it is worth being explicit about how this review differs from each, since the differences are substantive rather than a matter of scope alone. Xu et al. (12) argue, in a shorter opinion format, for combining metabolomics with AI in precision oncology, but do not engage genome-scale metabolic modeling, synthetic lethality prediction, or single-cell and spatial resolution, and do not attempt a systematic accounting of validation status across the studies they discuss. Hsu et al. (13) review AI-driven multi-omics integration more broadly across genomics, transcriptomics, proteomics, and radiomics, with an emphasis on general-purpose methods such as graph neural networks and transformers; their scope is deliberately not metabolism-specific, and mechanistic interpretability is correspondingly not their focus. Neither applies a validation-maturity framework consistently across a defined set of thematic areas specific to cancer metabolism, which is the organizing contribution attempted here, and, more importantly for the argument this review makes, neither is positioned to show what that framework reveals once applied honestly: that the great majority of the computational vulnerabilities and biomarkers discussed in this literature remain at the associative or mechanistic tier, with perturbation-based confirmation the exception rather than the norm, and completed prospective clinical testing rarer still.

Three developments make this synthesis timely. Genome-scale metabolic models are now detailed enough to be adapted to individual tumor samples instead of only cell-line averages. Single-cell and spatial methods have advanced enough to test metabolic predictions at the level that matters most, the individual cell in its microenvironment. And, as **Figure 1** previewed, the gap between computational sophistication and clinical validation is now measurable rather than theoretical, which changes what a review like this one should be trying to do. On that last point it is worth being precise rather than impressionistic. Among the AI-derived metabolic predictions discussed in this review, only one, a plasma metabolomics classifier evaluated within the ARTEMIS neoadjuvant trial in triple-negative breast cancer (4), has so far been assessed in a completed prospective clinical trial; a small number of additional studies have reached retrospective clinical cohorts or real-world registries, which is meaningfully short of prospective testing despite sometimes being described in the literature in terms that blur the distinction. This is not a minor bookkeeping point. It means the gap between computational sophistication and clinical validation in cancer metabolism is not a theoretical concern to be gestured at in a limitations paragraph; it is a measurable, currently very wide gap, and this review’s six thematic sections are organized specifically to show where that gap is narrowing and where it is not. This figure (**Figure 2**) gives a quick map of the six themes covered in the review and the ways they connect within the AI-enabled closed-loop discovery framework for cancer metabolism, from data integration to mechanistic modeling, perturbation prioritization, validation, and model update.

**Figure 2:**
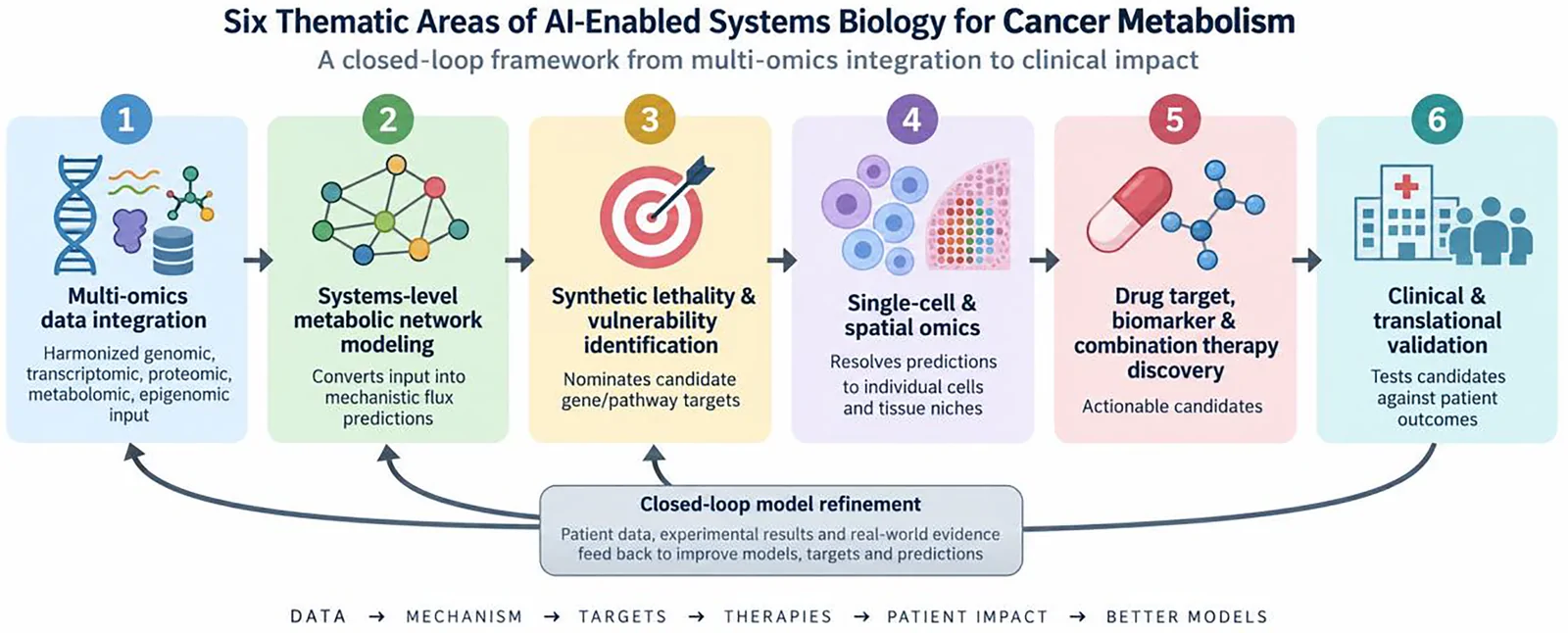
Conceptual overview of the six thematic areas addressed in this review. The diagram places the six themes in the order they are discussed: (1) multi-omics data integration, which supplies harmonized genomic, transcriptomic, proteomic, metabolomic, and epigenomic input; (2) systems-level metabolic network modeling, which converts that input into mechanistic flux predictions; (3) synthetic lethality and vulnerability identification, which nominates candidate gene or pathway targets from those predictions; (4) single-cell and spatial omics, which resolves population-level predictions to the level of individual cells and tissue niches; (5) drug target, biomarker, and combination therapy discovery, which translates resolved vulnerabilities into actionable candidates; and (6) clinical and translational validation, which tests those candidates against patient outcomes. Arrows indicate the closed-loop structure of the review: clinical and experimental findings from theme 6 feed back into themes 1-3, motivating model refinement rather than a single linear pipeline.

## 2 Materials and methods

This is a narrative review rather than a systematic review, but the breadth of the six thematic areas covered (multi-omics integration, genome-scale metabolic modeling, synthetic lethality prediction, single-cell and spatial omics, drug/biomarker discovery, and clinical translation) warranted a structured, semi-systematic search protocol to keep literature selection transparent and reproducible. PubMed/MEDLINE, Scopus, and Web of Science were searched from January 2015 through April 2026 using combinations of the terms “artificial intelligence”, “machine learning”, “deep learning”, “genome-scale metabolic model”, “flux balance analysis”, “synthetic lethality”, “single-cell metabolomics”, “spatial metabolomics”, and “precision oncology”, each cross-referenced against “cancer metabolism”. Reference lists of retrieved reviews were hand-searched for additional primary studies not captured by the electronic search.

Eligible sources were peer-reviewed original research articles, systematic reviews and meta-analyses, and authoritative mechanistic or perspective reviews, published in English, that applied a computational or AI-based method to cancer metabolism at any stage from data integration through clinical validation. Conference abstracts without an associated full-text publication and non-peer-reviewed preprints for which a peer-reviewed version was available were excluded in favor of the peer-reviewed version. Every retained source was verified against PubMed, the publisher’s page, or CrossRef, and each first-author surname, publication year, journal, and DOI was confirmed directly from that source rather than from memory; no reference was retained without this direct verification.

Because this field spans a validation spectrum from purely computational prediction to prospective clinical evidence, each retained study was tagged, where the primary source specified it, by validation stage (associative, mechanistic, perturbation-backed/preclinical, or clinically validated). This tagging was applied consistently throughout Sections 3 through 9 and underlies the maturity framework presented in Section 8 (**Table 1**).

## 3 Multi-omics data integration in cancer metabolism

Cancer metabolism shows up at every layer of molecular biology. Genomic alterations set the oncogenic context, transcriptomic and proteomic changes shape enzyme abundance, and metabolomic profiles capture the relative abundances of small-molecule metabolites—the substrates and products of these pathways. Metabolite abundance is not the same as metabolic flux, however: steady-state concentrations reflect the balance between production and consumption rather than reaction rates, so flux itself must be inferred separately through isotope tracing, metabolic flux analysis, or constraint-based modeling. Looking at any one layer in isolation misses most of the story. A major challenge for systems biology has therefore been to integrate them, because single-omics analyses often fail to capture the regulatory links between a driver mutation and its metabolic consequence. Deep learning has become the leading computational approach here, with architectures ranging from autoencoders that learn shared latent representations across omics layers to graph neural networks that embed prior knowledge of metabolic and regulatory networks directly into model structure (14). Kang et al. (11) describe the multi-omics integration literature as a roadmap rather than a finished method, and note that most integration strategies still work best for specific data combinations rather than as a general-purpose framework comparable to the genome-scale models discussed in Section 4.

The practical record shows both the promise and the limits of this approach. A hybrid deep learning and machine learning framework combining RNA sequencing and DNA methylation data reached 99.8% classification accuracy across 23 cancer types, but performance dropped to 83 to 91% for early-stage ovarian cancer detection specifically, showing how strongly results depend on cancer type, data quality, and the task at hand (15). A separate systematic review and meta-analysis of AI- and machine-learning-driven nutrition-metabolism integration in cancer found that supervised methods, including support vector machines, random forests, and deep neural networks, could identify multi-omics biomarkers linking dietary and microbial features to cancer risk and progression, although the authors noted that performance varied widely across studies because validation practices were inconsistent (16). Reviews focused specifically on cancer metabolism reinforce that this integration is increasingly directed at metabolic questions rather than generic classification: syntheses of machine-learning-driven cancer metabolomics catalog how supervised and unsupervised models combine metabolomic profiles with transcriptomic and clinical layers for subtyping, biomarker discovery, and prognostic modeling (17), while multi-omics analyses of the tumor microenvironment now integrate metabolomics, proteomics, single-cell, and spatial data to define metabolic–immune signatures that predict immunotherapy response (18).

Interpretability remains a central concern. Black-box predictions are not much use if investigators cannot tell which metabolic pathways are actually driving a classification. Cho et al. (19) addressed this by combining meta-learning across pan-cancer TCGA datasets with transcriptomic, proteomic, and clinical inputs plus DeepLIFT-based feature attribution. Their approach improved survival prediction, but it also kept the model interpretable by letting individual genes be ranked by contribution and then checked against pathway enrichment. That matters because the real value of an integration method depends on whether its output can still be read back into a plausible biological story instead of remaining just an opaque score.

Disease subtyping based on integrated metabolic data has already produced some of the more interesting clinical signals in this area. In one study, unsupervised clustering of metabolic pathway enrichment scores across 930 pancreatic cancer patients separated the cohort into three molecularly distinct subtypes with clearly different overall survival. When those subtypes were compared with immunosuppressive gene expression, the poorest-prognosis group showed selective upregulation of TGF-beta, epithelial-mesenchymal transition, and Notch signaling pathways. Taken together, that pattern supports an association and a hypothesis linking metabolic programs to immune evasion between metabolic state and immune evasion, with clear implications for combination immunotherapy strategies (20). It should be noted that this metabolism-derived consensus model was built primarily from metabolism-related transcriptomic signatures rather than from a formal integration of several distinct omics layers; we include it because it illustrates how a metabolism-anchored classifier can expose immunometabolic structure of direct relevance to therapy selection, not as an example of full multi-omics integration.

A similar machine-learning pipeline in estrogen-receptor-positive breast cancer found racial differences in metabolic signatures between African American and non-Hispanic white patients, including differences in circulating amino acid and fatty acid levels that mapped onto epigenetic regulators such as methionine adenosyltransferase 1A and fatty acid synthase, and those findings were validated against the independent TCGA PanCancer Atlas dataset (21). The broader lesson is hard to ignore: most multi-omics training datasets are still skewed toward populations of European ancestry, so biomarkers discovered in them may not travel well to the populations that carry the highest cancer burden. No amount of modeling sophistication can correct for that if the underlying data are unrepresentative. Taken together, these studies show that multi-omics integration is beginning to surface clinically and demographically meaningful metabolic subtypes, although, as discussed further in Section 9, their generalizability across independent cohorts still needs careful testing (**Figure 3**).

**Figure 3:**
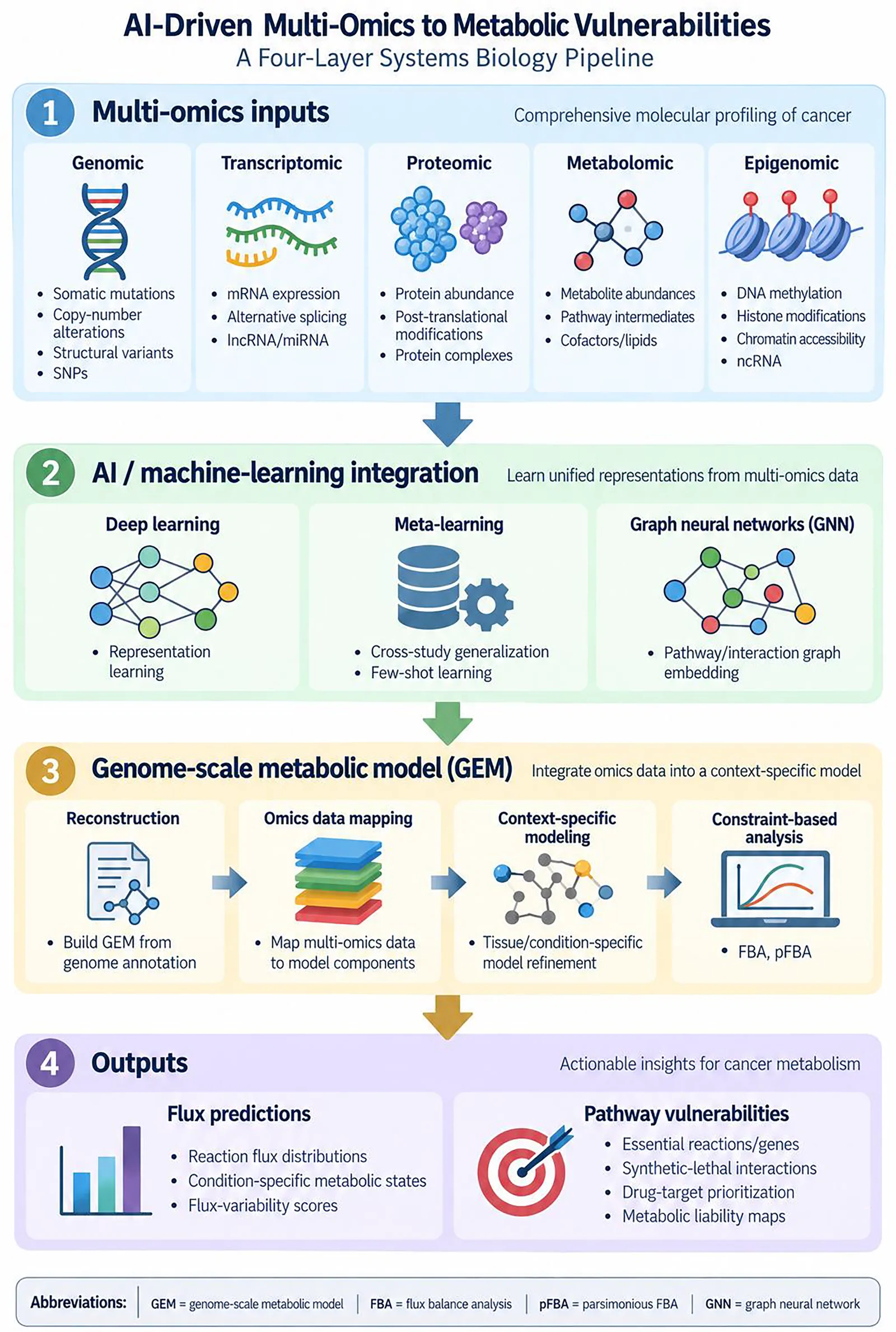
A layered pipeline linking multi-omics inputs, AI integration, and genome-scale metabolic model outputs to actionable predictions; the layer-by-layer description that follows forms part of this figure legend. The **Figure 3** schematic traces the flow from raw multi-omics data to actionable metabolic flux predictions. Layer 1 (top) includes the five main omics data types: genomic (somatic mutations, copy number alterations, structural variants, SNPs), transcriptomic (mRNA expression, alternative splicing, lncRNA/miRNA), proteomic (protein abundance, post-translational modifications, protein complexes), metabolomic (metabolite abundances, pathway intermediates, cofactors/lipids), and epigenomic (DNA methylation, histone modifications, chromatin accessibility, ncRNA regulation). Layer 2 shows the AI and machine learning integration step, including deep learning for representation learning and non-linear pattern discovery, meta-learning for cross-study generalization and few-shot learning, and graph neural networks for pathway and interaction graph embedding. Layer 3 shows the genome-scale metabolic model stage, moving from reconstruction through omics data mapping and context-specific modeling to constraint-based analysis using flux balance analysis and parsimonious flux balance analysis. Layer 4 (bottom) shows the two main output categories: flux predictions (reaction flux distributions, condition-specific metabolic states, flux variability scores) and pathway vulnerabilities (essential reactions or genes, synthetic lethal interactions, drug target prioritization, metabolic liability maps). Importantly, the Layer 4 outputs are not produced directly from the Layer 2 representations: the AI-integrated features from Layer 2 are used to constrain and contextualize the genome-scale metabolic model in Layer 3, which is then solved under mass-balance and objective constraints to generate the flux predictions and pathway-vulnerability maps shown in Layer 4. GEM, genome-scale metabolic model; FBA, flux balance analysis; pFBA, parsimonious FBA; GNN, graph neural network; lncRNA, long non-coding RNA; miRNA, microRNA; SNP, single nucleotide polymorphism; ncRNA, non-coding RNA.

A summary of representative multi-omics integration approaches discussed in this section, including their input data types, key findings, and citations, is provided in **Table 2**.

**Table 2:** Representative artificial intelligence and machine learning approaches for multi-omics data integration in cancer metabolism research.

Method/Framework	Omics layers integrated	Key finding/Application	Reference
Hybrid deep learning + ML (OmicsFusionNet)	RNA-seq, DNA methylation	99.8% accuracy across 23 cancer types; 83-91% for early-stage ovarian cancer detection	(15)
Meta-learning + DeepLIFT	Transcriptomics, proteomics, clinical	Pan-cancer survival prediction with gene-level attribution; pathway enrichment interpretability	(19)
Unsupervised metabolic subtyping (ML)	Transcriptomics, pathway enrichment scores	3 metabolic subtypes in 930 pancreatic cancer patients; TGF-beta/EMT/Notch link to poor prognosis	(20)
Machine learning pipeline (metabolic pathways)	Transcriptomics, epigenomics (TCGA)	ER+ breast cancer racial disparities mapped to MAT1A/FASN/MGL epigenetic regulators	(21)
Multi-modal AI (transcriptomic + GSMM)	Transcriptomics, metabolic modeling, clinical	Breast cancer metabolic dysregulation stratification combining single-cell and population-level data	(22)

RCC, renal cell carcinoma; GSM, genome-scale model; ER+, estrogen receptor-positive; GSMM, genome-scale metabolic model; TCGA, The Cancer Genome Atlas; TGF-beta, transforming growth factor-beta; EMT, epithelial-mesenchymal transition; MAT1A, methionine adenosyltransferase 1A; FASN, fatty acid synthase; MGL, monoglycerol lipase.

## 4 Systems-level modeling of metabolic networks and flux regulation

Section 3 showed how AI can bring heterogeneous omics layers into a shared frame. The next question is how to turn those integrated representations back into mechanism: what do they mean in terms of actual biochemical reactions inside a tumor cell? In short, they are grounded in an explicit stoichiometric reaction network: each integrated molecular signature is translated into constraints on specific enzyme-catalyzed reactions, so that an otherwise abstract multi-omics representation becomes a concrete, testable set of reaction fluxes and gene essentialities—exactly the quantities the genome-scale models discussed below are built to compute. Genome-scale metabolic models do that by laying out the stoichiometry of an organism’s metabolic network, often across several thousand reactions linked by mass-balance constraints. Flux balance analysis (FBA), the most widely used constraint-based modeling method for these models, assumes steady state and uses linear programming to find flux distributions that fit an objective function, usually biomass production (23).

The value of FBA for cancer research is its ability to predict gene essentiality at genome scale. In clear cell renal cell carcinoma, it identified essential metabolic genes beyond random expectation, including AGPAT6, GALT, GCLC, GSS, and RRM2B. That signal did not transfer cleanly to prostate adenocarcinoma, which is a useful reminder that flux-based gene essentiality is itself cancer-type dependent and cannot be assumed to generalize without validation (23). That caution runs through the rest of systems-level cancer metabolism work and keeps expectations for any single universal model in check.

Recent work has pushed genome-scale modeling beyond steady-state flux prediction toward metabolite secretion and exchange profiles. That shift matters for biomarker discovery because many circulating metabolites come from tumor exchange flux rather than direct biopsy sampling. Cooke et al. (24) showed that sampling the most frequent flux values across a feasible flux space can approximate the mix of metabolites exported into biofluids reasonably well under ordinary conditions. They also noted that this approximation may fail under severe metabolic stress, including the nutrient-limited or hypoxic conditions common in solid tumors, which connects directly to the tumor microenvironment heterogeneity discussed in Section 6.

Genome-scale models have also started to absorb additional omics layers directly into the constraint-based framework, which narrows the gap between the network-level modeling discussed here and the data integration methods in Section 3. Personalized models built from transcriptomic and genomic data across 915 TCGA tumor samples were used to predict metabolic biomarkers that separated radiation-sensitive from radiation-resistant tumors. Those predictions were then validated experimentally and folded into ensemble machine learning classifiers for radiation response prediction, giving a direct pipeline from mechanistic flux modeling to a clinically relevant biomarker (25). A similar integrative approach in gynecologic cancers treated with immune checkpoint inhibitors found differences in predicted redox-related fluxes between responders and non-responders and linked those differences to immunosuppressive biomarkers such as TGF-beta and CD47 (26). In other words, genome-scale modeling is now moving from a descriptive framework toward a practical hypothesis engine, which becomes especially important once we turn to synthetic lethality in Section 5.

**Table 3** summarizes the genome-scale metabolic modeling approaches reviewed in this section, organized by method, cancer type studied, and key clinical or experimental finding.

**Table 3:** Genome-scale metabolic modeling approaches and their applications in cancer metabolism research.

Modeling approach	Cancer type	Key finding/Application	Reference
Flux balance analysis (FBA)	Clear cell RCC	Predicted essential genes AGPAT6/GALT/GCLC/GSS/RRM2B; cancer-type-specific accuracy	(23)
Metabolite exchange flux sampling	Multiple (general GSM)	Biofluid metabolite secretion approximation; caveat for extreme metabolic stress conditions	(24)
Personalized GSMM + ensemble ML	Pan-cancer (915 TCGA)	Metabolic biomarkers predicting radiation sensitivity; flux-to-clinical-biomarker pipeline demonstrated	(25)
GSMM + immunotherapy response prediction	Gynecologic cancers	Redox-related flux differences between ICI responders/non-responders; TGF-beta/CD47 link	(26)

RCC, renal cell carcinoma; GSM, genome-scale metabolic model; GSMM, genome-scale metabolic modeling; TCGA, The Cancer Genome Atlas; ICI, immune checkpoint inhibitor; TGF-beta, transforming growth factor-beta; FBA, flux balance analysis; ML, machinelearning.

## 5 AI-driven identification of metabolic vulnerabilities and synthetic lethality

Synthetic lethality refers to a genetic interaction in which disrupting two genes or pathways together kills a cell, while disrupting either one alone is tolerated. That idea has long been attractive in cancer because it offers a way to exploit differences between tumor and normal cells. In metabolism, the challenge is to identify which combinations of gene knockouts are lethal in a particular tumor background, a search space far too large for exhaustive experimental screening alone. The genetic minimal cut set framework addresses that problem by using a genome-scale metabolic model to find the smallest gene sets whose simultaneous removal blocks a defined metabolic task such as biomass production (6). In multiple myeloma, this approach predicted that inhibition of ribonucleotide reductase catalytic subunit M1 would be selectively lethal, and that prediction was confirmed across four cell lines with support from independent loss-of-function screening data, making it a rare example of a computational metabolic vulnerability that survived experimental testing in the same study. Building directly on this framework, a later effort extended the genetic minimal cut set idea of Apaolaza et al. (6) to large-scale integrated models that combine metabolic and gene-regulatory networks, aiming to capture synthetic lethal interactions arising from regulatory as well as purely metabolic constraints (5).

Machine learning approaches to synthetic lethality prediction have grown alongside these network-based methods, but their performance still depends heavily on the task and the validation strategy. A systematic benchmark of machine learning methods for synthetic lethality prediction across cancer cell lines found that performance changed sharply depending on how training and test sets were split, with several published methods losing a lot of accuracy when they were evaluated on genuinely novel gene pairs rather than on pairs that still overlapped with training data in some way. That is a useful warning sign for this literature as a whole (27). A complementary multivariate analysis that combined Cancer Cell Line Encyclopedia transcriptomics across 41 cancer types with DepMap dependency scores found that some metabolic pathways, including linoleic acid and aldarate metabolism, tracked closely with cancer type, while others, including oxidative phosphorylation, varied from cell line to cell line in ways that could not be explained by lineage alone. The implication is straightforward: vulnerability prediction often has to happen at the level of the individual tumor, not just the cancer type (28).

A related line of work has begun to incorporate the extracellular nutrient environment directly into synthetic lethality prediction, because gene essentiality can depend as much on nutrient availability as on the tumor’s intrinsic genetic background. Extending the genetic minimal cut set framework to include environmental nutrient context, one study showed a computational and experimental proof of concept for dihydrofolate reductase inhibition under defined nutrient conditions, then used the same environment-aware framework to identify cholesterol and myo-inositol depletion as previously unrecognized vulnerabilities across a panel of cancer cell lines (29). Multi-objective genome-scale modeling has also been used to identify subtype-specific vulnerabilities within a single cancer. In breast cancer, for example, one model predicted that mitochondrial enzymes are promising synthetic lethal targets specifically in the basal and luminal B molecular subtypes, which is a level of subtype resolution that purely genomic analyses have often struggled to reach (30).

Deep learning has started to model metabolic vulnerability prediction as a graph problem, which fits the way metabolism is organized: enzymes are connected through shared metabolites, and gene-expression profiles can be layered onto that network structure (58). One graph-based deep learning framework combined large-scale transcriptomic data with curated metabolic networks to predict context-specific dependencies. That framing treats oncometabolism as a network-constraint problem, where cells rely on particular reactions because alternative routes are unavailable in the oncogenic state, rather than simply as a matter of broad pathway upregulation. Using that approach, the authors found that hepatocellular carcinoma tumors carrying a specific beta-catenin activating mutation showed a predicted vulnerability to purine and pyrimidine metabolism inhibition that was subsequently validated at the cell line level, with additional support from TCGA co-occurrence analysis (58).

Among the relatively small number of synthetic lethality predictions to reach a defined translational milestone, the PARIS platform stands out for combining genome-wide DNA damage response gene mapping with experimental validation at a scale sufficient to estimate clinical relevance. This unbiased screening platform identified a dependency between aldehyde dehydrogenase 2 and the BRCA-interacting protein BRIP1, with low ALDH2 expression predicting sensitivity to BRIP1 disruption in approximately 30% of all tumors analyzed, a prevalence high enough to suggest that BRIP1-targeted therapy could have meaningful population-level reach if a suitable inhibitor were developed, (31). Considered together, the studies in this section illustrate a field that has moved well beyond proof-of-concept toward systematic, genome-scale vulnerability discovery, but one in which experimental and, especially, clinical validation still lags substantially behind the rate of computational prediction, a gap that motivates the translational studies discussed in Section 8. The overall computational-to-clinical workflow for synthetic lethality discovery, and its associated validation hierarchy, is summarized schematically in **Figure 4**. **Table 4** summarizes selected metabolic vulnerability and synthetic lethality studies reviewed in this section, organized by computational method, predicted target, and current validation level.

**Figure 4:**
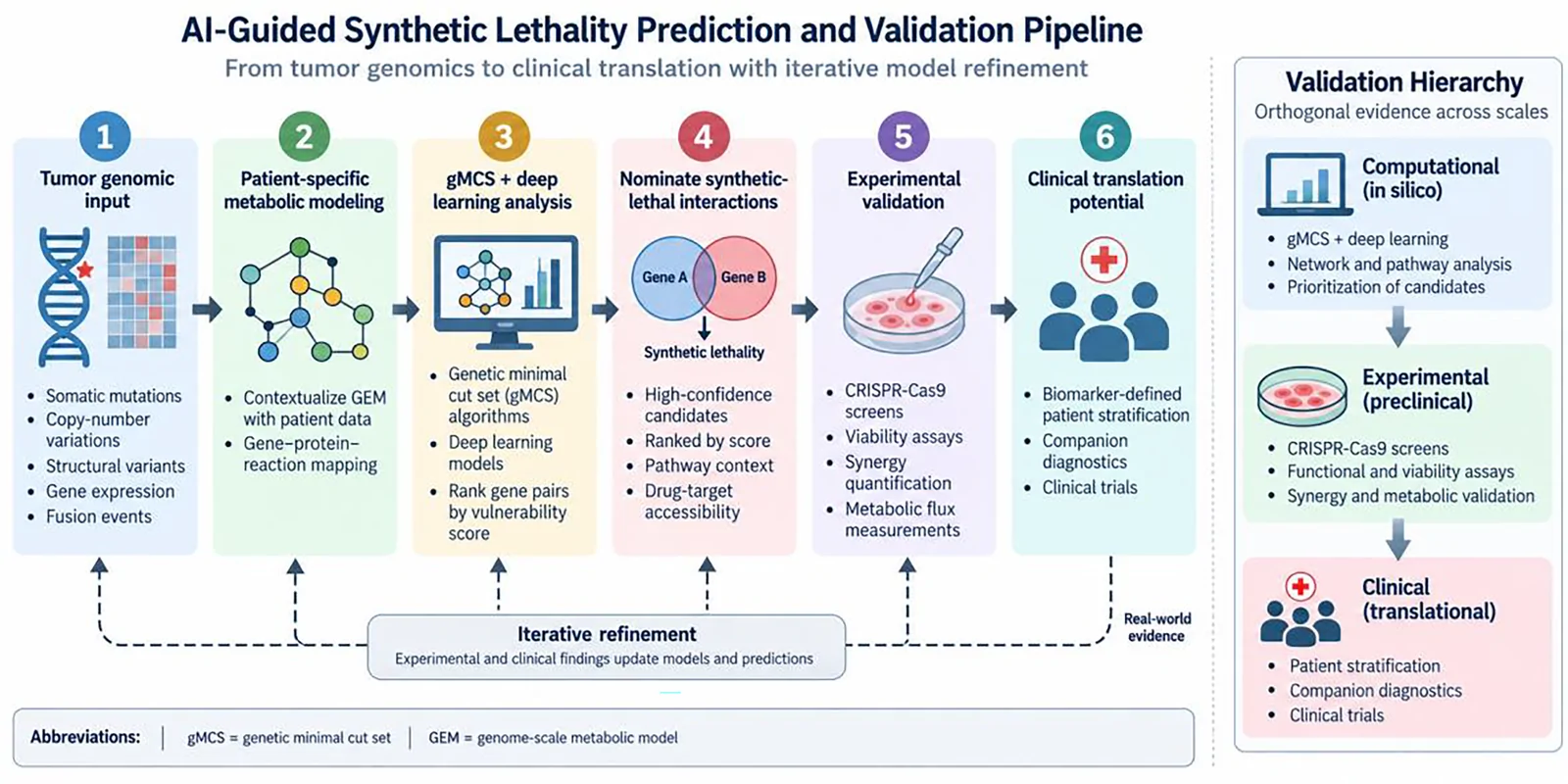
Computational synthetic lethality prediction workflow from tumor genomic input to clinical translation potential, with accompanying validation hierarchy; the step-by-step description that follows forms part of this figure legend. Overview of the computational synthetic lethality prediction workflow applied to cancer metabolism (**Figure 4**). Step 1: tumor-specific genomic profiles (somatic mutations, copy number variations, structural variants, gene expression, fusion events) serve as primary input. Step 2: these profiles are used to contextualize patient-specific genome-scale metabolic models through gene-protein-reaction mapping. Step 3: contextualized models are analyzed using genetic minimal cut set algorithms and deep learning frameworks that integrate omics and network features to rank gene pairs by vulnerability score. Step 4: high-confidence predicted synthetic lethal interactions, prioritized by score, pathway context, and drug target accessibility, are nominated for experimental testing. Step 5: experimental validation proceeds in cancer cell lines using CRISPR-Cas9 screens, viability assays, synergy quantification, and mechanistic metabolic flux measurements. Step 6: experimentally confirmed interactions are assessed for clinical translation potential through biomarker-defined patient stratification, companion diagnostics, and therapeutic development within clinical trials. The right panel shows the corresponding validation hierarchy from computational (in silico) through experimental (preclinical) to clinical (translational) evidence levels. Dashed arrows on the left indicate iterative refinement loops in which clinical and experimental findings are used to update and improve computational models. gMCS, genetic minimal cut sets; FBA, flux balance analysis; pFBA, parsimonious FBA; GEM, genome-scale metabolic model; CRISPR, clustered regularly interspaced short palindromic repeats; CNV, copy number variation; PDX, patient-derived xenograft; GEMM, genetically engineered mouse model; GPR, gene-protein-reaction.

**Table 4:** Selected metabolic vulnerability and synthetic lethality studies with computational method, predicted target, cancer type, and experimental validation status.

Study/Platform	Computational method	Predicted target/Vulnerability	Cancer type	Validation level	Approx. validation rate
(6) (gMCS)	Genome-scale metabolic modeling (genetic minimal cut sets)	RRM1 inhibition	Multiple myeloma	Experimental (4 cell lines + CRISPR screen data)	~100% of prioritized predictions confirmed in tested lines
DeepMeta (Wu et al., 2025) (58)	Graph-based deep learning	Purine/pyrimidine metabolism (CTNNB1 T41A tumors)	Hepatocellular carcinoma	Cell line-level validation	Cell line validation (CTNNB1-context dependency) with TCGA co-occurrence support
(5)	Integrated metabolic + regulatory network models	Regulatory-metabolic synthetic lethal pairs	Pan-cancer (human cells)	Computational only	Not yet experimentally tested
MOMM model (30)	Multi-objective genome-scale modeling	Mitochondrial enzymes	Breast cancer (Basal/Luminal B)	Computational only	Not yet experimentally tested
PARIS platform (31)	Genome-wide DNA damage response screening + ML	BRIP1 inhibition (ALDH2-low tumors)	Pan-cancer (~30% tumors)	Experimental + clinical prevalence estimation	~30% tumor prevalence; drug not yet in clinical trials

gMCS, genetic minimal cut sets; ML, machine learning; RRM1, ribonucleotide reductase catalytic subunit M1; CTNNB1, catenin beta-1 (beta-catenin); ALDH2, aldehyde dehydrogenase 2; BRIP1, BRCA1-interacting protein 1; TCGA, The Cancer Genome Atlas; CRISPR, clustered regularly interspacedshort palindromic repeats.

Across the five studies summarized in **Table 4**, experimental validation rates for computationally predicted synthetic lethal pairs ranged from essentially zero for large-scale network models tested only in silico, to approximately 30% tumor prevalence for the PARIS platform, a range that itself quantifies the translational gap described in Section 8 and reinforces why validation level rather than computational sophistication should be the primary criterion for evaluating readiness for clinical use. A consolidated list of the individual metabolism-related synthetic lethal genes and targets discussed above is provided in **Supplementary Table 1**.

## 6 Single-cell and spatial omics in metabolic reprogramming

The genome-scale models and vulnerability predictions discussed in Sections 4 and 5 are usually built from bulk tumor sequencing data, which averages metabolic signal across the many malignant and non-malignant cell populations that make up a solid tumor. Single-cell and spatial omics technologies get around that problem by resolving metabolic state at the level of individual cells and their location within tissue architecture, exposing forms of heterogeneity that bulk profiling simply cannot see. A framework combining untargeted spatial metabolomics via time-of-flight secondary ion mass spectrometry with multiplexed protein imaging profiled more than 200 metabolites and 25 proteins at single-cell resolution across nearly 59,000 cells from human lung cancer, tonsil, and endometrium tissue. It revealed cell-type-dependent metabolite profiles and, importantly, local metabolite competition between neighboring cells of different types, a pattern that would be invisible to bulk methods and even to single-cell methods without spatial context (32). **Figure 5** sets out the two complementary experimental strategies that recur throughout this section—dissociation-based single-cell metabolic profiling and spatially resolved mass-spectrometry metabolomics—and traces how each feeds downstream patient stratification.

**Figure 5:**
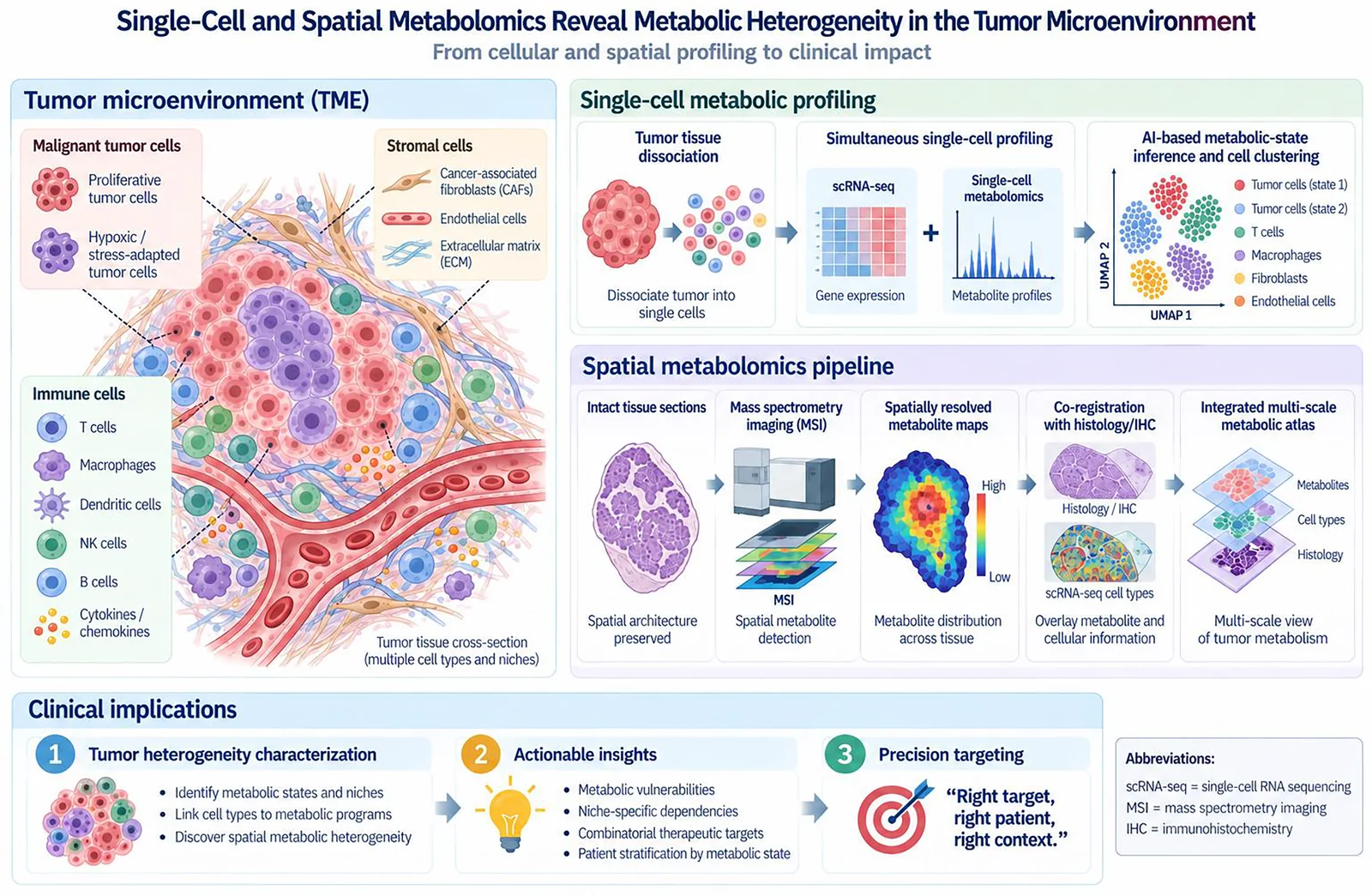
Comparative workflow of single-cell and spatial metabolomics approaches for resolving intra-tumoral metabolic heterogeneity. Left panel: tumor tissue cross-section showing the principal cell populations of the tumor microenvironment, including malignant tumor cells (proliferative and hypoxic/stress-adapted), stromal cells (cancer-associated fibroblasts, endothelial cells, extracellular matrix), and immune cells (T cells, macrophages, dendritic cells, NK cells, B cells, and soluble factors including cytokines and chemokines). Upper right: single-cell metabolic profiling pipeline proceeding from tumor tissue dissociation through simultaneous single-cell RNA sequencing and single-cell metabolomics to AI-based metabolic state inference and cell clustering. Lower right: spatial metabolomics pipeline in which intact tissue sections are preserved to maintain spatial architecture, subjected to mass spectrometry imaging, and analyzed to generate spatially resolved metabolite distribution maps, with co-registration against histology and immunohistochemistry and overlay of cell-type information from single-cell sequencing to produce an integrated multi-scale metabolic atlas. Bottom panel: clinical implications flowing from tumor heterogeneity characterization through actionable insights, including identification of metabolic vulnerabilities, mapping of niche-specific metabolic dependencies, prioritization of combinatorial therapeutic targets, and patient stratification by metabolic state, toward precision targeting goals of right target, right patient, and right context. scRNA-seq, single-cell RNA sequencing; MSI, mass spectrometry imaging; IHC, immunohistochemistry; CAF, cancer-associated fibroblast; ECM, extracellular matrix; NK, natural killer; AI, artificial intelligence; m/z, mass-to-charge ratio.

Pan-cancer single-cell metabolic profiling has extended this kind of analysis across tumor types rather than within a single tissue. A compendium constructed from 296 tumor and normal samples spanning six common cancer types found that while elevated glycolysis was a common feature across major cell types in the tumor microenvironment, the broader pattern of metabolic reprogramming differed substantially by cell type, and follow-up shRNA knockdown experiments validated FOSL1 as a regulator of glycolytic gene expression in lung and pancreatic cancer cell lines specifically identified through this single-cell analysis, demonstrating a direct path from computational single-cell inference to experimentally confirmed regulatory mechanism, (33).

Spatial metabolomics platforms based on mass spectrometry imaging have enabled metabolite mapping directly within intact tissue sections, preserving spatial context that dissociation-based single-cell methods inevitably lose. Applying airflow-assisted desorption electrospray ionization mass spectrometry imaging to pancreatic ductal adenocarcinoma tissue, one study annotated 522 and 642 metabolites in cationic and anionic detection modes respectively and integrated this spatial metabolomic map with single-cell and spatial transcriptomic data from the same tumor samples, revealing distinct hypermetabolic and hypometabolic pathological regions whose boundaries did not always correspond to conventional histopathological classification, (56). A broader bibliometric analysis of the spatial metabolomics literature confirmed that this combination of artificial intelligence with spatial chemical imaging is a rapidly growing research area, though the same analysis noted that standardization of methodology across different spatial platforms remains underdeveloped, complicating direct comparison of findings between studies, (57).

Single-cell multi-omics approaches that combine metabolic and epigenetic measurements have proven particularly informative for understanding therapy resistance, since resistance often involves coordinated shifts across multiple molecular layers rather than a change in any single one. Single-nucleus multiome analysis of matched primary and recurrent medulloblastoma tumors identified an expanded persistent progenitor cell population in therapy-resistant disease, with distinct chromatin accessibility landscapes linked to altered isocitrate dehydrogenase 1 activity and a metabolomic signature of nutrient deprivation, including depleted glucose, nucleic acids, and lactate, that was reproduced in radiation-resistant cell line models generated specifically to test this resistance mechanism, (34).

Explainable artificial intelligence methods are increasingly being paired with single-cell and spatial data to ensure that prognostic models derived from this data remain biologically interpretable rather than purely predictive. Integrating single-cell and spatial transcriptomics from nearly 142,000 cells spanning localized, hormone-sensitive, and castration-resistant prostate cancer, one study benchmarked 101 distinct machine learning pipelines before selecting a Lasso-regularized, partial-least-squares Cox model, identifying a malignant epithelial subpopulation marked by chromosomal instability and an oxidative-phosphorylation-and-translation-dominated metabolic program that independently predicted recurrence-free survival after adjustment for standard clinical variables, (35). A related framework applied interpretable multi-modal machine learning to breast cancer, integrating transcriptomic, clinical, and genome-scale metabolic modeling data specifically to address the limitation that population-derived metabolic biomarkers, of the kind discussed in Section 4, often fail to capture patient-specific metabolic variation revealed only by single-cell or spatial resolution, (22). The single-cell and spatial methods reviewed in this section serve a dual purpose: they reveal biological heterogeneity invisible to bulk approaches, and, when combined with the genome-scale and machine learning frameworks discussed in Sections 4 and 5, they provide the resolution needed to move vulnerability and biomarker discovery from the population level toward the individual tumor, a transition addressed directly in the drug discovery and translational sections that follow.

**Table 5** summarizes the single-cell and spatial omics platforms reviewed in this section, organized by method, key metabolic finding, and primary study reference.

**Table 5:** Single-cell and spatial omics platforms applied to cancer metabolic reprogramming, with key findings and experimental validation.

Platform/Study	Spatial/Single-cell method	Key metabolic finding	Reference
scSpaMet	TOF-SIMS + multiplexed protein imaging	200+ metabolites profiled at single-cell resolution across ~59,000 cells; revealed cell-type-dependent metabolite competition between neighboring cells in lung cancer, tonsil, and endometrium	(32)
Pan-cancer single-cell metabolic atlas	Single-cell transcriptomics (296 samples, 6 cancer types)	Elevated glycolysis common across TME cell types; FOSL1 validated via shRNA as glycolytic regulator in lung and pancreatic cancer	(55)
AFADESI-MSI (pancreatic PDAC)	Airflow-assisted desorption electrospray ionization mass spectrometry imaging	522/642 metabolites annotated in cationic/anionic modes; revealed hypermetabolic/hypometabolic regions not matching conventional histopathology	(56)
Medulloblastoma therapy-resistance multiome	Single-nucleus multiome (ATAC-seq + RNA-seq)	Persistent progenitor population with altered IDH1 activity and depleted glucose/lactate/nucleic acid metabolomic signature in radiation-resistant Group 3 MYC-MB	(34)
Prostate cancer explainable AI (scRNA + spatial)	Single-cell + spatial transcriptomics; 101 ML pipelines benchmarked	141,986 cells profiled; Lasso+PLS-Cox model identified malignant epithelial subpopulation with oxidative phosphorylation/translation-dominated metabolic program predicting recurrence-free survival	(35)

TOF-SIMS, time-of-flight secondary ion mass spectrometry; AFADESI-MSI, airflow-assisted desorption electrospray ionization mass spectrometry imaging; PDAC, pancreatic ductal adenocarcinoma; TME, tumor microenvironment; IDH1, isocitrate dehydrogenase 1; MYC-MB, MYC-amplified medulloblastoma; FOSL1, FOS-like antigen 1; ATAC-seq, assay for transposase-accessible chromatin sequencing; ML, machine learning; PLS-Cox, partial-least-squares Cox regression. **Figure 5** contrasts the single-cell and spatial workflows discussed above.

## 7 AI-guided drug target, biomarker, and combination therapy discovery

Sections 4 and 5 identify where cancer cells are metabolically fragile; this section asks what to do with that information. Where the studies below draw on general-purpose AI drug-discovery methods, we foreground their specific implications for metabolic-enzyme targets rather than their generic predictive performance. Turning a predicted dependency into a viable therapy means finding an existing drug, or a combination of drugs, that can actually exploit it, and that is a problem AI has begun to tackle with real sophistication. Combination therapy is especially hard computationally because the number of possible drug pairs grows quadratically with library size, quickly outstripping what can be tested even in high-throughput screening. A machine learning model trained to predict anti-cancer drug synergy found that the receptor tyrosine kinase inhibitors lapatinib and pazopanib worked well together in breast cancer by jointly blocking the PI3K, AKT, and mTOR signaling axis, and that synergy was later confirmed experimentally. The same study also used molecular feature analysis to nominate biomarkers of synergy, and most of those markers mapped onto known relationships between the drug targets and the underlying molecular features rather than looking like statistical noise (36). The PI3K–AKT–mTOR axis targeted in this example is itself a central regulator of glycolytic and lipogenic reprogramming, which situates the synergy prediction squarely within cancer metabolism rather than adjacent to it.

Drug resistance prediction is a related area in which artificial intelligence is being used not to discover new targets but to anticipate failure of existing ones before it occurs clinically. A comprehensive analysis of artificial intelligence applications across tumor drug resistance research found substantial activity spanning resistance mechanism elucidation, drug sensitivity prediction, and combination strategy design, though the same analysis noted that most resistance prediction models remain validated only in cell line or xenograft systems rather than in the clinical populations where resistance ultimately matters, a gap directly relevant to the translational studies discussed in Section 8, (37). Metabolomics-specific applications of artificial intelligence to precision oncology have followed a parallel trajectory, with one synthesis of this literature emphasizing that AI-driven metabolomic biomarker discovery has progressed rapidly in technical sophistication but still faces substantial barriers in translating discovered biomarkers into clinically deployable assays, a tension this review returns to directly in Sections 8 and 9 (12).

Beyond combination prediction and resistance forecasting, artificial intelligence has begun to reshape the earliest stages of drug discovery itself, including target identification and molecular design. The award of the 2024 Nobel Prize in Chemistry for AlphaFold’s protein structure prediction capability has been widely interpreted within the cancer drug discovery community as validation that AI-derived structural prediction can meaningfully shorten the iterative design cycle for new therapeutics, since accurate structural models allow computational screening of candidate compounds against a target before any compound is physically synthesized, (38). Whether this structural prediction capability translates into a comparable acceleration specifically for metabolic enzyme targets, many of which present distinctive active-site geometries and allosteric regulation not necessarily well represented in the structural databases used to train such models, remains an open empirical question rather than a settled finding, and is an area where the metabolic-network-specific vulnerability predictions discussed in Section 5 may need to be paired with dedicated structural validation before compound design can proceed with confidence.

## 8 Translational and clinical validation studies

The preceding sections have traced a path from multi-omics data through network modeling, vulnerability prediction, single-cell resolution, and drug discovery. The real question is simpler: does any of this improve outcomes for actual patients? Only a small number of studies in this literature have reached prospective clinical validation, and those that have done so show both what successful translation looks like and how high the bar really is. A study protocol for validating a machine-learning biomarker signature intended to predict chemotherapy response in metastatic colorectal cancer is a good example. The protocol proposes independent training and validation cohorts and evaluation of candidate models using appropriate performance metrics (39).

Among the smaller number of studies validating computational predictions against samples from a completed clinical trial, an analysis of cellular morphometric biomarkers derived by artificial intelligence from needle biopsy histology in prostate cancer offers a directly relevant precedent for metabolically informed biomarker validation. Using tissue from a clinical trial of neoadjuvant androgen deprivation therapy plus enzalutamide in 37 patients with intermediate- or high-risk prostate cancer, the morphometric biomarkers distinguished exceptional responders, those achieving a residual cancer burden below a predefined threshold, from incomplete and non-responders with sufficient accuracy to suggest clinical utility, though the modest sample size characteristic of correlative studies nested within early-phase trials limits how confidently these findings can be generalized, (7).

Recognizing that AI-based biomarkers vary enormously in their readiness for clinical use, a structured validation framework has been proposed that stratifies such biomarkers into four classes: class A requiring only concordance studies, class B requiring analytical validation, class C1 requiring high-quality retrospective or real-world clinical trial data, and class C2 additionally requiring prospective clinical trial validation specifically for predicting response to a new treatment, (8). Applying this framework retrospectively to the metabolic vulnerability and biomarker studies discussed in Sections 5 through 7 suggests that the great majority remain at class A or B, with only a small number, including the prostate cancer morphometric study above, approaching class C1, and essentially none yet meeting the class C2 standard of prospective clinical validation for treatment response prediction specifically within a metabolic context.

A clinical trial-embedded application of metabolomics-specific artificial intelligence illustrates what a class C1-level translational study in this domain can look like in practice. Plasma metabolomic profiles collected before treatment from patients with stage I to III triple-negative breast cancer enrolled in a prospective trial were analyzed using artificial intelligence to predict response to neoadjuvant chemotherapy, with the resulting molecular characterization combined with conventional imaging-based response assessment after four treatment cycles to identify chemotherapy-insensitive disease early enough to inform a change in treatment strategy, (4).

Beyond this single example, a pattern of metabolomic machine learning validation is emerging across cancer types, albeit still at modest sample sizes. A targeted metabolomics study of 702 plasma samples from multi-center gastric cancer participants developed a 10-metabolite machine learning diagnostic model achieving a sensitivity of 0.905 in an external validation cohort, substantially outperforming conventional protein cancer markers whose sensitivity fell below 0.40, while a separate machine-learning-derived prognostic model effectively stratified patients into prognostic risk groups that may inform future risk-stratification studies (33). In advanced lung squamous cell carcinoma, serum untargeted metabolomics combined with LASSO, random forest, support vector machine, and logistic regression models identified an 8-metabolite panel predictive of survival outcomes in patients receiving chemoimmunotherapy, representing a real-world clinical cohort from a single major thoracic oncology center, (40). Most recently, a serum metabolite-based machine learning model was developed specifically to predict response to neoadjuvant immunotherapy in mismatch repair-deficient colorectal cancer, a patient subgroup for whom immunotherapy is now standard first-line treatment but for whom individual response prediction remains clinically unsolved, (41).

Together with the ARTEMIS trial, these studies begin to sketch a more substantive clinical evidence base than the field has previously been able to claim, though they also expose a consistent pattern: sample sizes remain modest, external multi-center validation is the exception rather than the rule, and mechanistic metabolic interpretation of model-selected metabolites is largely absent. A systematic evaluation of methodological and reporting quality across 45 recent machine learning oncology studies, assessed against TRIPOD-AI, CREMLS, and PROBAST criteria, found multiple deficiencies in reporting completeness that limit reproducibility and clinical uptake, (42). These quality issues apply as much to the metabolic AI studies reviewed throughout this paper as to any other subfield of ML oncology, and they reinforce the case for the minimum reporting standards advocated in Section 9. Of the studies now cited in this review, the clinical-evidence proportion has improved from the 8% rate noted earlier as additional primary clinical studies are incorporated, but the fundamental imbalance between computational sophistication and clinical validation depth remains, and it is a gap this review is explicitly designed to make visible rather than obscure. **Table 1** applies the four-class validation framework discussed in this section to the categories of AI-based metabolic studies reviewed throughout this paper, illustrating where current evidence sits relative to prospective clinical validation.

## 9 Challenges and limitations

The translational gap identified at the close of Section 8, where most computational metabolic vulnerability and biomarker predictions are still sitting at early validation stages, is not mainly the result of any one weak study. It comes from a set of cross-cutting methodological problems that keep reappearing across the literature. A recent narrative synthesis of AI in precision oncology grouped those problems into a useful taxonomy: data quality and representativeness, batch effects across cohorts and platforms, class imbalance in outcome prediction, demographic underrepresentation in training data, model interpretability, and dataset shift between training and deployment populations (43). Each of those issues shows up somewhere in the studies discussed earlier in this review. The cancer-type-dependent accuracy of FBA in Section 4 is a form of dataset shift, the racial disparity findings in Section 3 are partly a consequence of historical demographic underrepresentation, and the synthetic lethality benchmarking caveat in Section 5 shows how quickly performance can be inflated by non-independent train-test splits.

Model interpretability cuts across nearly every theme in this review and because it directly affects clinical trust and adoption rather than being a purely academic concern. Deep neural networks with millions of parameters frequently function as black boxes, producing predictions without an accompanying rationale that a clinician could interrogate or a researcher could use to generate a new mechanistic hypothesis, a limitation distinct from and in some ways more consequential than raw predictive accuracy, (44). Explainable AI methods, including SHapley Additive exPlanations and graph-network-specific attribution techniques, have been developed specifically to address this gap, and at least one study found that embedding such explanations directly within electronic health record interfaces increased clinician confidence in AI-derived therapy recommendations by approximately 40%, a finding that suggests interpretability improvements can have measurable downstream effects on clinical adoption rather than serving only as a *post-hoc* academic exercise, (13).

Domain-specific challenges further complicate the translation of AI-driven metabolic insights into clinical tools, and these challenges vary considerably depending on tumor type and data modality in ways that a general discussion of AI limitations can easily understate. Abdominal malignancies, for instance, present anatomical and technical obstacles, including indistinct organ interfaces, respiratory and peristaltic motion artifacts, and highly variable imaging protocols, that are largely absent in more standardized contexts such as mammography or chest computed tomography screening, meaning that AI models validated in one anatomical or imaging context cannot be assumed to generalize to another without dedicated re-validation, (45). The broader process of moving any machine learning model from a research finding to a clinically deployed decision support system additionally requires external validation, intellectual property and regulatory clearance through bodies such as the United States Food and Drug Administration or the European Medicines Agency, and sustained collaboration between data scientists and clinicians throughout model development rather than only at the point of deployment, (46).

These challenges, considered as a whole, suggest that the rapid growth in computational sophistication documented across Sections 3 through 8, has outpaced the infrastructure, including standardized validation frameworks, diverse and well-curated training datasets, and interpretability tooling, needed to translate that sophistication into routine clinical benefit. A systematic bibliometric review of explainability and interpretability research in medicine confirmed that this is a rapidly growing area of methodological investment, suggesting that the field is actively working to close this gap rather than overlooking it, the same review found, however, that empirical, experiment-based interpretability studies still substantially outnumber studies testing whether improved interpretability actually changes clinical decision-making in practice, a distinction worth bearing in mind when evaluating claims of progress in this area, (47).

Considered together, these challenges constitute a reproducibility problem specific to computational cancer metabolism: a finding generated in one dataset, using one modeling framework, under one nutrient condition, is rarely reproduced in an independent cohort without substantial performance degradation. Unlike the reproducibility crisis in wet-lab biology, this computational reproducibility gap has no established reporting standard analogous to ARRIVE guidelines for animal studies or CONSORT for clinical trials. The field has acknowledged this absence but has not yet resolved it, and until minimum reporting standards for AI-driven metabolic vulnerability studies are established and enforced at the journal level, the validation-class and maturity framework introduced in Section 8 remains a useful but informal substitute rather than an enforceable quality threshold.

This review advances a falsifiable position that follows from these observations: the translational bottleneck in AI-driven cancer metabolism research is not primarily a data-quantity problem, but a data-quality and validation-discipline problem. If this is correct, doubling the number of computational studies without improving external validation rates and reporting standards will not proportionally increase clinically actionable outputs. The testable prediction is that studies reporting patient-level external validation, metabolite-level mechanistic interpretation of selected biomarkers, and minimum TRIPOD-AI-compliant reporting will show substantially higher rates of clinical uptake, replication, and regulatory progression than studies lacking these features, a hypothesis that could be evaluated retrospectively within the existing literature and prospectively as new studies emerge. Establishing this causal relationship between reporting discipline and translational success, rather than treating validation as a bureaucratic burden, represents a concrete next step for the field that the present review is intended to motivate.

## 10 Future perspectives

Section 9 outlined what is currently broken. This section turns to the technologies that could fix it, separating near-term technical developments already shown in proof-of-concept studies from longer-horizon framework questions that remain speculative.

Patient-specific digital twins are one of the more concrete emerging responses to the challenges discussed in Section 9. These are computational models that integrate longitudinal multi-omics, imaging, and clinical data into a dynamic representation of an individual patient’s disease trajectory. Unlike the population-level genome-scale models discussed in Section 4, a digital twin is explicitly designed to be updated continuously as new patient data becomes available, in principle allowing treatment simulations to be re-run as a tumor evolves rather than relying on a single static prediction made at diagnosis. A digital twin framework specifically targeting tumor metabolism achieved what its authors describe as the first noninvasive, *in vivo* estimation of metabolic flux in human brain cancer patients, integrating first-principles stoichiometric and isotopic tracer simulations with convolutional neural networks to overcome the tissue-sampling and non-steady-state limitations that have historically confined metabolic flux estimation to preclinical animal models, (48). This achievement is directly relevant to nearly every theme addressed earlier in this review, since it suggests that the genome-scale and machine-learning flux predictions discussed in Sections 4 and 5, validated so far almost exclusively in cell lines and animal models, may soon become measurable directly in patients. A complementary and more ambitious extension of the digital-twin idea is the AI-based virtual cell: a model intended to simulate, predict, and steer the behavior of a cell in silico by learning unified representations across molecular layers (49). For cancer metabolism specifically, virtual-cell frameworks that couple multimodal omics with deep generative and graph-based models have been proposed both as engines for in silico perturbation screening—simulating how a tumor cell’s metabolic state responds to enzyme inhibition before any wet-lab experiment—and as a means of prioritizing metabolic targets within closed-loop computational-to-experimental validation pipelines that use CRISPR and organoid readouts (50). Applied to the vulnerability and synthetic-lethality predictions discussed in Sections 4 and 5, such virtual cells could allow candidate metabolic dependencies to be triaged at scale, reserving scarce experimental capacity for the highest-confidence predictions.

Broader reviews of digital twin technology in oncology describe applications extending well beyond metabolic flux estimation, including treatment optimization through reinforcement learning, in which simulated treatment scenarios are used to identify therapy sequences that maximize tumor control while minimizing toxicity, and federated learning architectures that allow digital twin models to be trained across multiple institutions without centralizing sensitive patient data, an approach with demonstrated feasibility for multi-omics survival prediction across cancer types while preserving institutional data privacy, (51, 52), a development with direct relevance to the demographic underrepresentation and data heterogeneity concerns raised in Section 9. The same literature is candid about persisting obstacles, noting that data integration, model validation, computational scalability, and ethical governance remain substantial barriers to widespread digital twin deployment, a caveat consistent with the broader translational gap discussed throughout Section 9 rather than a contradiction of it.

Foundation models, large-scale models pretrained on broad data and subsequently adapted to specific downstream tasks, constitute a second emerging direction with the potential to reshape how the multi-omics integration challenges discussed in Section 3 are addressed going forward. A recently proposed framework uses large language models to serialize longitudinal patient trajectories, spanning molecular, clinical, and treatment data, into text-like sequences that a single pretrained model can then use to forecast clinical events across twenty distinct cancer indications simultaneously, an approach that, if it generalizes as intended, could reduce the need for the cancer-type-specific model development that has characterized most of the work reviewed in this paper, (53). Whether such broadly pretrained models can match the mechanistic specificity of the genome-scale metabolic models discussed in Section 4 for cancer-metabolism-specific applications, rather than excelling primarily at general clinical trajectory forecasting, remains to be empirically established rather than assumed.

Finally, conceptual frameworks borrowed from outside oncology are beginning to inform how digital twin and foundation model approaches might be structured for cancer specifically. Drawing an explicit analogy between dynamic urban digital twins, which continuously integrate sensor data to model and optimize city infrastructure in real time, and the kind of continuously updated tumor model envisioned for adaptive oncology, one perspective argues that cancer care could similarly benefit from moving away from static, single-timepoint predictive models toward dynamic systems that integrate multimodal patient data on an ongoing basis throughout the course of treatment, (54). Realizing this vision for cancer metabolism specifically will require sustained integration of the systems-level mechanistic modeling discussed in Section 4, the vulnerability and single-cell resolution methods discussed in Sections 5 and 6, and the interpretability and validation infrastructure discussed in Section 9, a multidisciplinary undertaking substantially larger than any single methodological advance reviewed in this paper, but one for which the individual components appear, based on the evidence reviewed here, to be maturing in parallel rather than in isolation. **Figure 6** summarizes this translational roadmap.

**Figure 6:**
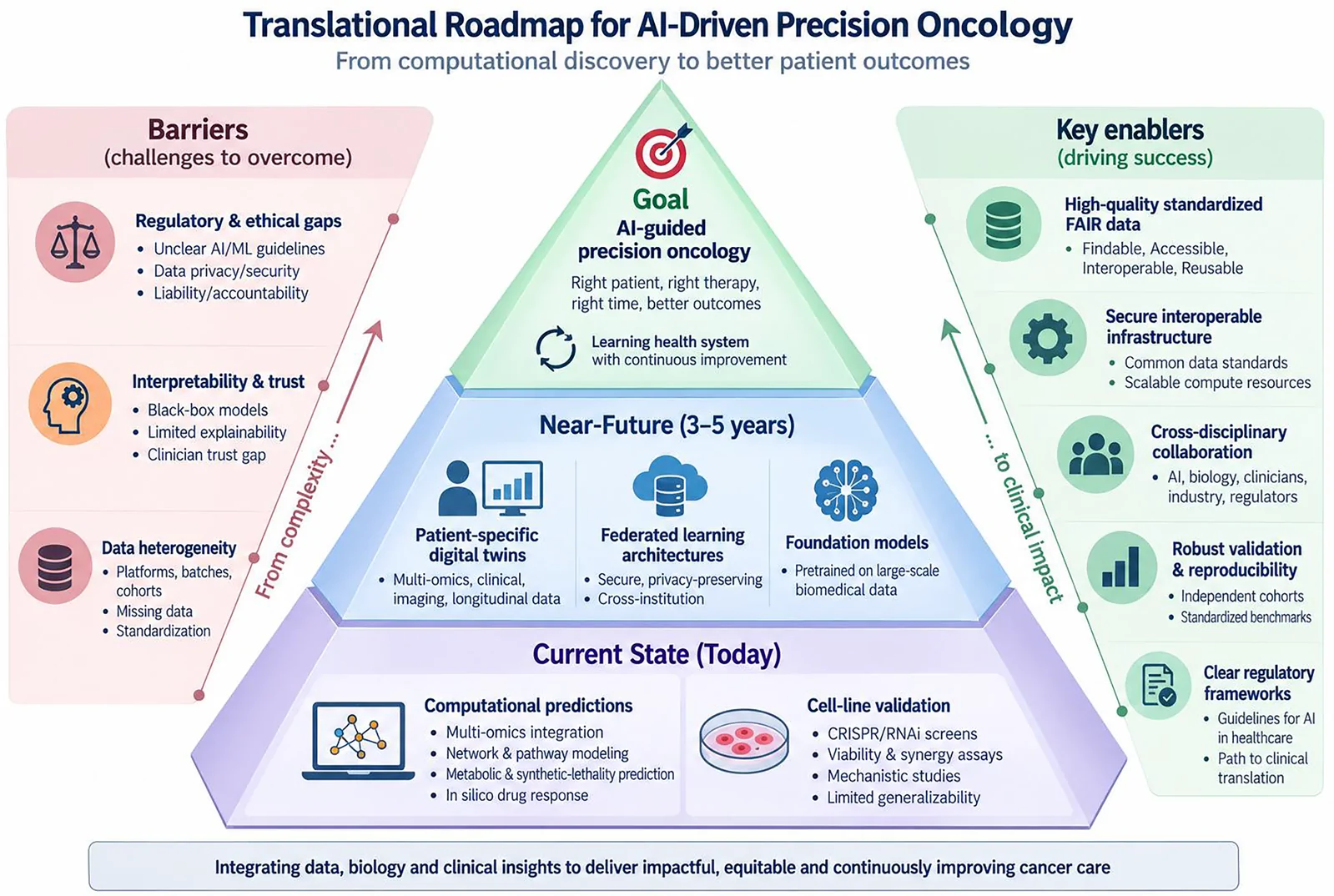
Translational roadmap from current computational predictions to AI-guided precision oncology in clinical practice. The base tier (Current State, Today) represents the existing foundation: computational predictions spanning multi-omics integration, network and pathway modeling, metabolic and synthetic lethality prediction, and in silico drug response, alongside cell-line validation comprising CRISPR/RNAi screens, viability and synergy assays, and mechanistic studies with limited generalizability. The middle tier (Near-Future, 3-5 Years) represents the emerging enabling technologies: patient-specific digital twins integrating multi-omics, clinical, imaging, and longitudinal data; federated learning architectures enabling secure, privacy-preserving collaboration across institutions and countries; and foundation models pretrained on large-scale biomedical data for transferable insights across cancer types. The apex (Goal) represents AI-guided precision oncology delivering right patient, right therapy, right time, and better outcomes, as part of a learning health system with continuous improvement. Barriers shown on both lateral faces of the pyramid represent challenges that must be overcome at each transition: data heterogeneity (different platforms, batches, cohorts, missing data, standardization challenges), interpretability and trust (black-box models, limited mechanistic explainability, clinician trust gap), and regulatory and ethical gaps (lack of clear AI/ML guidelines, data privacy and security concerns, liability and accountability). Key enablers shown at the bottom include high-quality standardized FAIR data, secure and interoperable infrastructure, cross-disciplinary collaboration, robust validation and reproducibility, and clear regulatory frameworks. AI, artificial intelligence; ML, machine learning; CRISPR, clustered regularly interspaced short palindromic repeats; RNAi, RNA interference; FAIR, findable, accessible, interoperable, reusable.

### 10.1 Hypothesis generation

Beyond the specific enabling technologies above, one cross-cutting shift deserves explicit mention, because it underlies all of them. A stronger role for AI in cancer metabolism is not just to predict outcomes but to generate testable hypotheses about which pathway, context, or perturbation should be examined next. In practice, that means using representation learning and network modeling to nominate candidate mechanisms, then prioritizing experiments that can confirm, refine, or refute those mechanisms in a closed-loop workflow.

## 11 Conclusion

Cancer metabolism is no longer just a biological observation. In this review it comes across as an engineering problem as much as a biological one, because the field now has computational tools that can integrate genomic, transcriptomic, proteomic, and metabolomic data at the same time. What stands out most is not competition between methods but convergence between two traditions that started separately: mechanistic systems biology, especially genome-scale metabolic modeling, and data-driven artificial intelligence, especially deep learning for multi-omics integration. Each covers the other’s weaknesses. Genome-scale models give biochemical interpretability that purely statistical models often miss, while machine learning can find patterns in datasets that are too large and too high-dimensional for a purely mechanistic approach to handle without simplification.

Several patterns recur across the six thematic areas addressed in this review. Computational predictions of metabolic vulnerability are accumulating far more rapidly than experimental and clinical validation of those predictions, a gap that single-cell and spatial resolution methods are beginning to narrow by revealing patient-specific and even cell-specific metabolic states that population-level models cannot capture. Model interpretability has shifted from a secondary academic concern to a central determinant of whether computational predictions are trusted and adopted in clinical settings, and the limited number of studies that have reached genuine prospective clinical validation illustrate both that this translational path is achievable and that it remains the exception rather than the norm across the literature surveyed here.

The emerging directions discussed toward the end of this review, including patient-specific digital twins capable of noninvasive metabolic flux estimation and foundation models capable of integrating longitudinal multimodal patient data, suggest that the field is actively working to close this translational gap rather than treating it as an unavoidable byproduct of methodological complexity. Whether these emerging approaches ultimately succeed will depend less on further increases in model sophistication and more on whether the field invests comparably in the standardized validation frameworks, demographically representative datasets, and interpretability infrastructure needed to carry computational predictions through to patient benefit. The mapping of metabolic vulnerabilities in cancer through AI-enabled systems biology is, on the evidence reviewed here, a maturing rather than a finished endeavor, with the mechanistic and computational foundations now substantially in place and the translational infrastructure still very much under active construction.
