# Supplementary material for: Identification of metabolic vulnerabilities and synthetic lethal targets utilizing artificial intelligence-driven precision medicine
Source: Front Oncol. 2026 Oct 27;16:1972698. doi: 10.3389/fonc.2026.1972698 (PMC13749214; doi:10.3389/fonc.2026.1972698)
Supplement: Supplementary file 1 [file Table1.doc]

**Supplementary Table 1.** *Metabolism-related synthetic lethal genes and targets discussed in Section 5, with the interacting gene or context, associated metabolic pathway, cancer type, and current validation level.*

| **Gene / target** | **Interacting gene or context** | **Metabolic pathway** | **Cancer type** | **Reference (validation level)** |
| --- | --- | --- | --- | --- |
| RRM1 (ribonucleotide reductase M1) | gMCS-predicted essentiality | Deoxyribonucleotide synthesis | Multiple myeloma | Apaolaza et al., 2017 [ref. 6] (experimental, 4 cell lines) |
| Purine / pyrimidine metabolism enzymes | CTNNB1 (β-catenin) T41A context | Purine and pyrimidine metabolism | Hepatocellular carcinoma | Wu et al., 2025 (cell line-level) |
| DHFR (dihydrofolate reductase) | Defined nutrient environment | Folate / one-carbon metabolism | Pan-cancer cell lines | Apaolaza et al., 2022 [ref. 29] (proof of concept) |
| Cholesterol biosynthesis and myo-inositol pathway genes | Environment-aware gMCS | Sterol / inositol metabolism | Pan-cancer cell lines | Apaolaza et al., 2022 [ref. 29] (computational) |
| Mitochondrial enzymes | Subtype-specific (multi-objective GSMM) | Mitochondrial / oxidative metabolism | Breast (basal, luminal B) | Gao et al., 2022 [ref. 30] (in silico) |
| ALDH2 (aldehyde dehydrogenase 2) | BRIP1 (synthetic lethal partner) | Aldehyde detoxification / DDR-linked | Pan-cancer (~30% prevalence) | Benfatto et al., 2021 [ref. 31] (experimental + prevalence) |

***Abbreviations:*** *ALDH2, aldehyde dehydrogenase 2; BRIP1, BRCA1-interacting protein C-terminal helicase 1; CTNNB1, catenin beta 1; DDR, DNA damage response; DHFR, dihydrofolate reductase; gMCS, genetic minimal cut sets; GSMM, genome-scale metabolic model; RRM1, ribonucleotide reductase catalytic subunit M1. Reference numbers correspond to the reference list of the main article.*
